# A randomised, double-blind, placebo controlled cross-over study to determine the gastrointestinal effects of consumption of arabinoxylan-oligosaccharides enriched bread in healthy volunteers

**DOI:** 10.1186/1475-2891-11-36

**Published:** 2012-06-01

**Authors:** Gemma E Walton, Congyi Lu, Isabel Trogh, Filip Arnaut, Glenn R Gibson

**Affiliations:** 1 Department of Food and Nutritional Sciences, University of Reading, Reading, RG6 6AP, United Kingdom; 2 Puratos Group, Industrialaan 25, Zone Maalbeek, 1702, Groot-Bijgaarden, Belgium

**Keywords:** Prebiotic, Arabinoxylan-oligosaccharides, Bifidobacteria, Butyrate, Intestine, Faecal, Human gut microbiota

## Abstract

**Background:**

Prebiotics are food ingredients, usually non-digestible oligosaccharides, that are selectively fermented by populations of beneficial gut bacteria. Endoxylanases, altering the naturally present cereal arabinoxylans, are commonly used in the bread industry to improve dough and bread characteristics. Recently, an *in situ* method has been developed to produce arabinoxylan-oligosaccharides (AXOS) at high levels in breads through the use of a thermophilic endoxylanase. AXOS have demonstrated potentially prebiotic properties in that they have been observed to lead to beneficial shifts in the microbiota *in vitro* and in murine, poultry and human studies.

**Methods:**

A double-blind, placebo controlled human intervention study was undertaken with 40 healthy adult volunteers to assess the impact of consumption of breads with *in situ* produced AXOS (containing 2.2 g AXOS) compared to non-endoxylanase treated breads. Volatile fatty acid concentrations in faeces were assessed and fluorescence *in situ* hybridisation was used to assess changes in gut microbial groups. Secretory immunoglobulin A (sIgA) levels in saliva were also measured.

**Results:**

Consumption of AXOS-enriched breads led to increased faecal butyrate and a trend for reduced iso-valerate and fatty acids associated with protein fermentation. Faecal levels of bifidobacteria increased following initial control breads and remained elevated throughout the study. Lactobacilli levels were elevated following both placebo and AXOS-breads. No changes in salivary secretory IgA levels were observed during the study. Furthermore, no adverse effects on gastrointestinal symptoms were reported during AXOS-bread intake.

**Conclusions:**

AXOS-breads led to a potentially beneficial shift in fermentation end products and are well tolerated.

## Background

Dietary approaches to manipulate the human gut microbiota have long been used as an approach to improve host health. The aim of probiotic and prebiotic inclusions into the diet are to increase beneficial gut bacteria and their activities, thus generating benefits to human health. These benefits include protection from gastroenteritis by pathogen inhibition [1], an improved tolerance to lactose [2], toxins [3] and cholesterol reduction [4], vitamin synthesis [3], improved mineral bioavailability [5], potential protection from bowel cancer [6-8], reduced symptoms of irritable bowel syndrome [9], improved digestion, gut function [10] and immune regulation [11].

Much interest in the development of prebiotics has been focused on non-digestible oligosaccharides. The prebiotic effects of fructooligosaccharides (FOS), inulin [12-15] and galactooligosaccharides (GOS) [9,16] have been extensively evidenced by changes in gut microbial composition through numerous volunteer trials. Despite the prebiotic oligosaccharides on the market, the development of novel forms of oligosaccharides as prebiotics is required to enable incorporation into more versatile food forms. In the bakery industry, the enzyme endoxylanase has been used for more than 30 years. Endoxylanase can improve dough consistency by enhancing strength of the gluten network [17]; improving bread volume and bread crumb structure through solubilising arabinoxylans (AX) [18]. Furthermore, AX have a beneficial influence on the bread-making process [18]. By using the appropriate type and dose of endoxylanases during bread-making, naturally present AX fibres can be converted into arabinoxylan-oligosaccharides (AXOS) fragments, with an average degree of polymerization (avDP) between 5 and 50. This provides an *in situ* production approach that may be considered as an alternative to straightforward fortification [18,19].

AXOS have shown prebiotic potential [20], leading to a potentially beneficially shift in gut bacteria in *in vitro* intestinal models [21,22], poultry studies [23] and human studies [24]. In intervention studies with poultry, it was observed that AXOS added to the diet dose dependently lead to reduced *Salmonella* numbers following infection when compared to a control group [25]. The positive impact of AXOS on intestinal fermentation was observed in a human study, whereby a daily intake of 10.0 g AXOS added to the diet of healthy persons was well-tolerated and promoted bifidobacterial growth while suppressing the excretion of urinary *p*-cresol, a potentially harmful metabolite of protein fermentation [24]. Additionally, Cloetens et al. [26] indicated that consumption of AXOS added to the diet at doses as low as 2.2 g was associated with a beneficial shift from urinary to fecal N excretion.

*In vitro* AXOS fermentation led to decreases in the potentially negative proteolytic products (phenol and p-cresol) in distal regions of a colonic simulator model, whilst increasing levels of short chain fatty acids [22]. A potentially favourable metabolic shift in fermentation was also observed in human volunteers [26]. Furthermore, the fermentation of AXOS has been linked to increased butyrate levels in rat studies [27]. Butyrate is a major energy source for intestinal epithelial cells and is considered to be a potential anti-cancer agent through its ability to stimulate apoptosis [28]. As such, its increase is largely regarded to be of benefit to the host.

Recently, there has been interest in the ability of the bacteria in the intestine to influence host immune functions, including through an up-regulation of immuno-globulins (Ig), that aid the fight against infection. It has recently been observed that probiotics can lead to increased salivary secretory IgA levels [29].

The current human trial was conducted to assess effects of the consumption of breads with *in situ* produced AXOS on the faecal microbiota and their fermentation products, such as faecal volatile fatty acid concentrations. A secondary aim was to determine whether AXOS-breads could impact on salivary secretory IgA concentrations. Volunteer diaries were collected to assess gastrointestinal effects such as tolerance, changes in bowel habit and food consumption and also emotional changes.

## Methods

### Production and characterisation of the investigational products

Refined wheat endosperm flour bread without *in situ* produced AXOS (control bread) was prepared by mixing 25000 g wheat flour (white) (Surbi, Dossche Mills & Bakery, Deinze, Belgium) with 500 g (2%) of salt, 500 g of baker’s yeast (Algist Bruggeman, Gent, Belgium), 500 g of endoxylanase-free bread improver mix (Puratos Group, Groot-Bijgaarden, Belgium), 500 g of gluten (Syral, Aalst, Belgium) and 14500 ml of water. Wheat/rye bread without *in situ* produced AXOS (placebo bread) was prepared by mixing 18750 g wheat flour (white) with 4375 g rye whole meal (Type 1740; Plange Mühle, Düsseldorf, Germany), 1875 g rye bran (Paniflower, Gent, Belgium), 500 g of salt, 500 g of baker’s yeast, 500 g of endoxylanase-free bread improver mix, 500 g of gluten and 15525 ml of water.

Wheat/rye bread with *in situ* produced AXOS (AXOS-bread) was prepared as placebo except that the water was reduced (15500 ml) and an endoxylanase preparation with an activity of 42000 Units/g (25 g) (Puratos Group, Groot-Bijgaarden, Belgium) was added. Major constituents of the breads are shown in Table 1.

**Table 1:** Major constituents of the breads used in the study

	**Wheat flour**	**Rye flour**	**Rye bran**	**Endoxylanase**
Control bread	100%	0%	0%	none
Placebo bread	75%	17.5%	7.5%	none
AXOS-bread	75%	17.5%	7.5%	42000 units/g

After mixing, the dough was divided in pieces of 820 g each, rounded manually and allowed to rise at room temperature for 20 min, followed by mechanical moulding, panning and fermentation proofing (90 min at 35 °C and 95% relative humidity). Doughs were baked in a hot air oven at 230 °C for 35 min. Two and a half hours after baking, the breads were sliced, packaged in polypropylene bags in rations of 180 g per pack, and frozen at −20 °C until consumption by the volunteers.

The breads were labelled by colours, therefore volunteers and investigators were unaware which bread treatment was issued.

### Compositional analyses of breads

Bread slices from the middle of the loaf were dried (12 h at 105 °C) then cooled to room temperature in an exsiccator for 1 h. The dried bread was homogenised to yield a powder. For characterisation of the AX population, aqueous extracts of breads dried and ground (2 g) was mixed with water (20 ml) and Termamyl 120 L (120 μl) (Novozymes, Bagsvaerd, Denmark), the Termamyl was pretreated for 1 h at 90 °C prior to use in order to destroy possible enzyme activities within, other than amylase. The suspension was incubated whilst shaking (37 °C, 30 min) and subsequently centrifuged (3000 x *g*, 4 °C, 15 min). The supernatant was stored at −20 °C until further analysis.

Total hydrolysable carbohydrate content of breads and total and reducing end saccharide contents of aqueous extracts thereof were measured by gas chromatography (GC) of alditol acetates obtained after acid hydrolysis, reduction and acetylation of the resulting monosaccharides as described by Courtin et al. [30]. Alditol acetates (1.0 μL) were separated on a Supelco SP-2380 polar column (30 m, 0.32 mm i.d.; 0.2 ím film thickness) (Supelco, Bellefonte, PA) in an Agilent chromatograph (Agilent 6890 series, Wilmington, DE) equipped with autosampler, splitter injection port (split ratio 1:20), and flame ionization detector. The carrier gas was helium. Separation was at 225 °C, and injection and detection were at 270 °C. The coefficient of variation of the results of this analysis was < 5,0%.

The total AX content of bread was calculated as described by Damen et al., 2012, [31] using concentrations of arabinose, galactose, xylose. As the anhydroxylose and anhydroarabinose units in AX are hydrated upon hydrolysis, a correction for this molecular mass shift has been incorporated in the calculations. Total AX in baseline/control, placebo and AXOS-bread were 2.0, 4.2 and 4.2% dry matter (dm), respectively. Endoxylanase treatment in AXOS-bread resulted in an AXOS level of 2.0% (dm) with an avDP of 18 while baseline/control and placebo bread had a water-extractable AX content of 0.6 and 0.9% (dm) with an avDP of 157 and 174, respectively. Seven slices of AXOS-bread (180 g/d) resulted in an intake of 2.2 g AXOS/day.

### Subjects

Volunteers were aged 18–55 years, BMI: 18.5-30 kg/m², of good general health and free of chronic diseases such as gastrointestinal illness and were required to avoid consumptions of any pharmaceuticals active on the gastrointestinal tract, including antibiotics, for at least 6 months before the study began. Volunteers were asked to refrain from any consumption of prebiotics and probiotics or laxatives within 1 month of the start and during the trial. Volunteers were excluded if pregnant or lactating.

Volunteers with a history of alcohol or drug abuse and current smokers were excluded as were those that had taken experimental drugs or been involved in experimental drug studies within the last month and those regularly consuming excessive alcohol (weekly more than 21 units/wk (male), 14 units/wk (female)). Volunteers were required to not have taken part in pre- and probiotic studies in the previous 3 months. Major surgery and physical or mental conditions that might effect participation in the study were also used as exclusion factors.

Furthermore, volunteers with severe allergy to foods including celiac disease; severe abnormal drug reactions, chronic gastroenterological complaints, those on calorie restricted or other special diets (e.g. Atkins diet, montignac diet) 6 weeks prior to the start of the study, those who had been vaccinated against with the current season’s influenza and AH1N1 flu, those with severe allergies, asthma and dermatitis and those who had had colonic irrigation within the previous 3 months were excluded from the study.

Forty-four healthy adults, 22 males and 22 females, were recruited from the local Reading area (UK) for participation in this study. Four subjects dropped out during the first feeding period, 3 due to the bread quantity, 1 to altered travel plans. A final volunteer dropped out (male) mid-way through the study for personal reasons. For analysis purposes, 40 volunteers were included, 1 treatment was completed by the final drop-out volunteer for the remaining time-points missing data was omitted from the calculations using Minitab 16 (Lead technologies inc., North Carolina, USA). The final study group of 40 volunteers consisted of 20 males and 20 females. Their mean age was 31.4 years (± 8.9) and average BMI was 23.3 kg/m2 (± 2.8). The Research Ethics Committee of The University of Reading approved the study and it was conducted in accordance with the Helsinki Declaration of 1975 as revised in 1983.

### Study design

**Figure 1:**
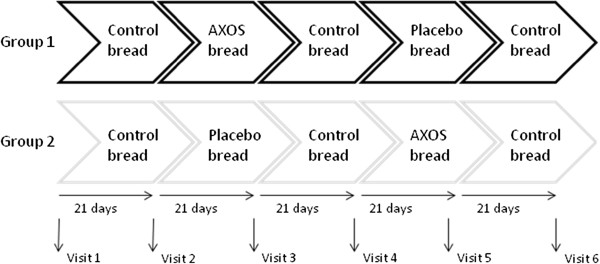
Cross-over trial design.

The dietary intervention study was a randomised, double-blind, placebo controlled cross-over trial. The dietary intervention was split into 5 periods including AXOS-treatment and placebo periods which were separated by 3 control periods, each period consisting of 21 days (Figure 1). The volunteers were randomly allocated into 1 of the 2 groups. Group 1 (n 20, 10 males and 10 females, Age 31.8 ± 9.9 years, BMI 23.2 ± 2.79 kg/m²) consumed control breads for the first period, then consumed AXOS-breads (treatment 1) for the following 21 days, and thereafter control breads for a further 21 days as a washout period. Placebo breads (treatment 2) were consumed for 21 days, followed by 21 days washout period of control bread. Meanwhile, Group 2 (n 20, 10 males and 10 females, Age 31.5 ± 8.5 years, BMI 23.2 ± 2.97 kg/m²) underwent the same control and treatment periods as Group 1, but they received placebo breads when the AXOS-breads were consumed by Group 1 and vice versa.

Volunteers were required to consume 7 slices of bread each day of the trial (total 180 g/day). Faecal and saliva samples were collected from each volunteer at 6 different time points, the first sample was prior to any intervention this was the baseline sample. Thereafter samples were taken post intervention period and post washout periods these sample periods are indicated in Figure 1.

### Volunteer diaries

To record gastrointestinal symptoms, volunteers kept diaries on a daily basis during the study. The diaries detailed stool frequency and consistency, abdominal pain, intestinal bloating and flatulence. Emotional changes including energetic status, happiness, alertness and stress levels were recorded as compared to normal. Lastly, concomitant medication, adverse events and failure to consume breads was also documented by the volunteers. A food frequency diary was completed by the volunteers over 4 consecutive days during the study periods (one day of which was during a weekend). Daily nutritional intake was calculated using Dietplan 6 (Forestfield software, West Sussex, United Kingdom).

### Sample collection and preparation of faecal sample

Freshly voided faecal samples were collected in plastic pots, placed in an anaerobic workstation (H2:CO2:N2, 10:10:80 by volume at 37 °C) (Don Whitley Scientific, West Yorkshire, UK) and processed within 2 h of voiding. Samples were diluted 1:10 (w:w) in phosphate-buffered saline (PBS, 0.1 M; pH 7.4), then homogenised using a stomacher (460 paddle beats/min) for 2 min. One ml of this was taken, centrifuged for 10 min at 13,000 x g at room temperature and the supernatant retained for volatile fatty acid analysis. Samples were vortexed with 3 mm glass beads (VWR) for 30 s before being centrifuged at 400 x g for 2 min at room temperature. The supernatant (375 μl) was fixed in 4% (w:v) (1125 μl) paraformaldehyde for 4 h at 4 °C. To wash the cells out of paraformaldehyde, samples were centrifuged at 13000 x g in 1 ml PBS for 5 min at room temperature and this centrifugation was repeated two more times, then samples were re-suspended in 150 μl PBS and stored in ethanol (1:1 by v:v) at −20 °C for fluorescence *in situ* hybrdidsation (FISH).

### Enumeration of faecal microbial population

FISH, using fluorescently labelled 16 S rRNA targeted oligonucleotide probes (Sigma-Aldrich, Steinheim, Germany) labelled at the 5’ end with the fluorescent dye Cy3, was used for bacterial enumeration of the faecal samples. The details of probes used in this study and hybridisation conditions are in Table 2. The FISH procedure was conducted as by Martìn-Peláez et al., 2008 [32].

**Table 2:** Hybridisation conditions for oligonucleotide probes used in this study

**Probe name**	**Sequence (5’ to 3’)**	**Hybridisation pre-treatment**	**Formamide (%) for hybridisation**	**Temperature (°C)**		**Bacteria covered**	
				**Hybridisation Washing**			
Ato291	GGTCGGTCTCTCAACCC	Lysozyme	0	50	50	*Atopobuim- Coriobacterium*	[33]
Bac303	CCAATGTGGGGGACCTT	None	0	46	48	*Bacteroides – Prevotella*	[34]
Bif164	CATCCGGCATTACCACCC	Lysozyme	0	50	50	*Bifidobacterium* spp	[35]
Chis150	TTATGCGGTATTAATCTYCCTTT	None	0	50	50	*Clostridium histolyticum- Clostridium perfringens*	[36]
Erec482	GCTTCTTAGTCARGTACCG	None	0	50	50	*Eubacterium rectale- Clostridium coccoides*	[36]
Lab158	GGTATTAGCAYCTGTTTCCA	Lysozyme	0	50	50	*Lactobacillus- Enterococcus*	[37]
Eco1531	CACCGTAGTGCCTCGTCATCA	None	35	37	37	*Escherichia coli*	[38]
Rrec584	TCAGACTTGCCGYACCGC	None	0	50	50	*Roseburia* and *Eubacterium rectale*	[39]
Fprau645	CCTCTGCACTACTCAAGAAAAAC	Lysozyme	15	46	48	*Faecalibacterium prausnitzzi*	[40]
EUB338‡	GCTGCCTCCCGTAGGAGT	None	35	46	48	Total bacteria 1	[41]
EUB338II‡	GCAGCCACCCGTAGGTGT	None	35	46	48	Total bacteria	[41]
EUB338III‡	GCTGCCACCCGTAGGTGT	None	35	46	48	Total bacteria	[41]

### Faecal volatile fatty acid analysis

Volatile fatty acids (VFA) were converted into tertbutyldimethylsilyl derivatives and analysis performed using the extraction procedure of Richardson et al., 1989 [42]. A HP 5890 series II GC system (Hewlett Packard, Palo Alto, Calif.) with a fused silica dimethyl polysiloxane 10 m, 0.18 mm ID, 0.20 μm df (Crossbond, Restek, Bellefonte, PA) was used. Injector and detector temperatures were 275 °C with the column temperature programmed from 60 °C for 3 min to 150 °C at 10 °C/min. Helium was the carrier gas. Injections of 1 μl volume were made by an autosampler. The response factors of compounds were calculated compared to the internal standard solution. This was used along with the calibrated compounds to ascertain concentrations. To maintain calibration, at every 15 samples, an external standard solution with known concentrations of volatile fatty acids was injected.

### Collection of saliva samples and sIgA assessment

Volunteers arrived at the Department following 30 min of fasting and no brushing of teeth. Volunteers were provided with a tube (50 ml, polypropylene, Corning, Amsterdam, Netherlands) in which to passively dribble for 2 min. Saliva samples were centrifuged at 13000 x g for 5 min at room temperature and the supernatant was stored at −20 °C. The sIgA concentration was assessed by an enzyme-linked immunosorbet assay (ELISA) kit according to the manufacturer’s instructions (Immundiagnostik AG, Bensheim, Germany). Absorption was detected at 450 nm against 630 nm using an ELISA reader (Tecan Genios, Switzerland). A range of IgA standards were used in every assay, for which a calibration curve was constructed, to quantify the sIgA amount within the samples.

### Statistical analyses

Bacterial numbers were statistically evaluated after transformation to log counts using Microsoft Office Excel 2007 (Washington, USA). Target bacterial groups, fermentation characteristics, salivary sIgA and faecal pH were grouped according to which treatment was consumed, then analysed using a paired t-test Minitab 16 (Lead technologies, North Carolina, USA). The bacteriology data was observed to follow a normal distribution pattern, except for in the case of the *E. coli* group analysis. Furthermore, the VFA data also followed non-normal distribution, therefore Wilcoxon signed rank was used for these analyses. In order to conduct the Wilcoxon signed rank test in a paired manner, changes in parameter were calculated and compared to a test-median of 0. Data is reported ± standard deviation.

To analyse the volunteer diaries volunteer responses were assigned scores, for example for with stool consistency −1 refers to soft; 0 to formed and 1 hard. These values for each volunteer over the intervention periods were averaged and paired t-tests used on these values. The dietary diaries were analysed using Dietplan 6 (Forestfield software, West Sussex, UK) during the placebo and AXOS intervention periods. Results were averaged and compared using a paired t-test. When a value of P < 0.05 was obtained a change was considered to be a statistically significant different.

By the use of a statistical power calculation, it was observed that at significance level of 5% (one-sided) that, a log change of 0.32 can be detected at a power of 90% with 39 volunteers. This calculation is based on the assumption that the within patient standard deviation is 0.42, which was as observed by Tuohy et al., 2001 [12].

## Results

In terms of faecal bacteriology, consumption of the initial control breads led to a significant increase in bifidobacteria from baseline (p = 0.0011). The bifidobacteria levels remained elevated throughout the study (Table 3).

**Table 3:** **Faecal bacterial numbers (log**_**10**_**cells/g faeces) determined by fluorescent*in situ*hybridisation for forty volunteers over the trial period during which they were consuming “placebo” or “AXOS” experimental bread products enriched with AXOS (mean values and standard deviations)**

				**baseline**		**control bread**			**pre- placebo treatment**			**placebo treatment**			**post- placebo washout**			**pre- AXOS treatment**			**AXOS treatment**			**post- AXOS washout**		
***Bifidobacterium***	9.14	±0.57	9.37	±0.59	^a^	9.32	±0.58	^a^	9.31	±0.44	^a^	9.33	±0.33	^a^	9.39	±0.40	^a^	9.40	±0.42	^a^	9.34	±0.40	^a^
***Clostridium histolyticum*group**	8.35	±0.72	8.43	±0.69		8.07	±0.64		8.04	±0.76	^a^	8.05	±0.85	^a^	8.14	±0.79		8.13	±0.74		8.14	±0.57	
***E. coli***	7.20	±0.39	7.35	±0.56		7.38	±0.47	^a^	7.33	±0.40		7.34	±0.45		7.34	±0.53		7.34	±0.50	^a^	7.40	±0.39	^a^
***Lactobacillus – Enterococcus***	8.85	±0.54	8.92	±0.37		8.60	±0.66		8.95	±0.55	^b^	8.72	±0.75		8.74	±0.69		9.05	±0.40	^bc^	8.69	±0.71	
***Atopobium*‒*Coriobacterium*group**	9.73	±0.24	9.80	±0.27		9.79	±0.28		9.84	±0.34		9.87	±0.26	^a^	9.88	±0.28	^a^	9.79	±0.21		9.87	±0.26	^a^
***E. rectale*group**	9.96	±0.27	9.87	±0.33		9.91	±0.33	^c^	10.07	±0.29	^ab^	10.08	±0.29	^a b^	9.99	±0.33		10.01	±0.34		10.08	±0.32	^a^
**Total bacteria**	10.73	±0.22	10.69	±0.22		10.70	±0.25		10.72	±0.20		10.68	±0.22		10.72	±0.18		10.81	±0.21	^b^	10.72	±0.25	
***Bacteroides***	9.54	±0.32	9.64	±0.44		9.85	±0.46	^ac^	10.07	±0.25	^ab^	10.11	±0.29	^a b^	9.91	±0.42	^ac^	9.99	±0.34	^abc^	10.12	±0.22	^a b^
***Roseburia – Eubacteria***	9.65	±0.21	9.69	±0.26		9.57	±0.44		9.79	±0.39	^ab^	9.67	±0.51		9.74	±0.27		9.73	±0.32		9.61	±0.47	
***Faecalibacterium prausnitzii*cluster**	9.57	±0.42	9.48	±0.44		9.53	±0.40		9.65	±0.33	^b^	9.52	±0.46		9.50	±0.51	^c^	9.56	±0.38		9.67	±0.41	^b^

A paired *t*-test was used to compare bacteria levels at different time points, for *E. coli* the non parametric Wilcoxon signed rank test was used, as this data did not follow a normal distribution.

a - different to baseline; b- different to pre-treatment; c- different to washout, (p < 0.05).

Lactobacilli and bacteroides significantly increased following both placebo (p = 0.018, <0.001) and AXOS-breads (p = 0.025, 0.050) as compared to pre- treatment levels. Post-AXOS treatment, bacteroides numbers continued to rise (p = 0.004) whilst lactobacilli numbers declined to pre-AXOS treatment levels (p = 0.007).

Bacteria in the *E. rectale* group, the *Roseburia – Eubacterium* subgroup and *Faecalibacterium prausnitzii* group increased significantly following placebo treatment, compared to pre-placebo treatment (p = 0.003; 0.010 and 0.044 respectively). *E. rectale* group and bacteroides both remained elevated following the post-placebo treatment washout, so were significantly different to pre-placebo treatment (p = 0.001; 0.001). Total bacteria increased significantly following AXOS treatment (p = 0.038).

Butyrate levels increased significantly following AXOS treatment (p = 0.041) (Table 4). Propionate levels were significantly higher post-AXOS treatment washout compared to pre-AXOS treatment (p = 0.045). Valerate levels significantly increased following consumption of control breads (p = 0.008) compared to baseline and remained elevated throughout all interventions, therefore the valerate concentration was significantly greater at pre-placebo, placebo, pre-AXOS and AXOS treatment compared to baseline (p = 0.010, 0.013, 0.004 and 0.017, respectively). Following AXOS-bread consumption, a trend for reduced iso-valerate concentration was observed (p = 0.058).

**Table 4:** Volatile fatty acid (VFA) concentrations (μmol/g faeces) determined by tertbutyldimethylsilyl derivatisation and GC analysis for forty volunteers over the trial period during which they were consuming control, placebo or experimental bread products enriched with AXOS. (Mean values and standard deviations)

	**baseline**		**control bread**		**pre- placebo treatment**		**placebo treatment**		**post- placebo washout**		**pre- AXOS treatment**		**AXOS treatment**		**post- AXOS washout**	
Acetate	45.6	±27.9	47.3	±21.8	51.1	±18.8	54.2	±26.4	48.0	±22.5	46.7	±22.3	55.1	±34.5	52.1	±19.6
Propionate	17.8	±13.7	16.5	±11.1	18.2	±10.1	19.5	±11.2	17.1	±10.9	16.1	±10.9^c^	17.5	±10.4	18.8	±8.6^b^
Butyrate	11.5	±7.9	13.6	±9.4	14.9	±10.1	14.6	±7.2^a^	15.0	±10.1	12.8	±8.8	16.3	±12.3^ab^	14.5	±7.9
Total acetate, propionate and butyrate	74.9	±44.7	77.4	±37.0	84.2	±33.3	87.8	±6.2	80.2	±38.3	75.6	±36.9	88.9	±52.4	85.4	±31.6
Valerate	1.2	±1.4	2.3	±2.3^a^	2.4	±2.3^a^	2.2	±1.8^a^	2.0	±2.3	2.4	±1.5^a^	2.3	±2.2^a^	1.7	±2.0
Iso-valerate	1.3	±0.6	1.3	±1.0	1.5	±1.3	1.3	±1.1	1.7	±1.1	1.4	±0.7	1.1	±1.0	1.7	±2.0
Caproate	0.8	±0.8	0.7	±0.9	0.9	±1.3	1.0	±1.4	0.8	±0.8	0.8	±0.8	0.7	±0.8	1.1	±1.4
Iso-butyrate	1.7	±0.7	1.6	±1.2	1.9	±1.5	1.7	±1.3	1.8	±1.2	1.6	±0.9	1.3	±1.1	1.8	±1.3
Total valerate, iso-valerate, caproate and iso-butyrate	4.9	±2.5	5.8	±4.5	6.7	±5.0	6.2	±4.9^a^	6.2	±3.7	6.1	±2.8	5.4	±4.2	6.3	±4.5

**P-values of VFA changes.** Wilcoxons signed rank test was used to determine statistical differences between levels of VFA, as this data did not follow a normal distribution.

a - different to baseline; b- different to pre-treatment; c- different to washout, (p < 0.05).

Furthermore, a trend for increased combined acetate, propionate and butyrate following AXOS breads compared to pre-AXOS treatment (p = 0.072) and decreased combined iso-valerate, iso-butyrate, valerate and caproate (p = 0.091) was observed.

Analysis of the volunteer diaries revealed that following placebo treatment there was an increase in stool number, compared to pre-placebo treatment (p = 0.049). Volunteers noted having more energy than normal following the placebo treatment compared to pre-placebo treatment (p = 0.006) (Table 5).

**Table 5:** Volunteers diaries detailing bowel habits and general mood on a daily basis throughout the study

	**control bread**		**pre-placebo treatment**		**placebo treatment**		**post- placebo treatment**		**pre-AXOS treatmen**t		**AXOS treatment**		**post- AXOS treatment**	
N^o^ of stools	1.30	±0.72	1.33	±0.75	1.43	±0.76^b^	1.44	±0.78	1.37	±0.76	1.39	±0.71	1.39	±0.79
Abdominal discomfort score *	0.11	±0.18	0.10	±0.16	0.10	±0.18	0.10	±0.17	0.13	±0.20	0.18	±0.37	0.10	±0.17
bloating score *	0.18	±0.27	0.18	±0.24	0.20	±0.27	0.17	±0.23	0.19	±0.27	0.25	±0.40	0.19	±0.23
flatulence score *	0.52	±0.53	0.47	±0.50	0.54	±0.55	0.49	±0.49	0.53	±0.53	0.51	±0.52	0.40	±0.48
Happy score **	0.01	±0.20	0.02	±0.17	0.02	±0.16	0.01	±0.18	0.01	±0.19	0.02	±0.14	0.00	±0.13
Alert **	−0.01	±0.15	−0.01	±0.09	0.03	±0.19	−0.01	±0.07	−0.01	±0.14	−0.01	±0.17	−0.01	±0.08
Energetic **	−0.07	±0.26	−0.07	±0.18	0.01	±0.14 ^b^	−0.01	±0.21	−0.03	±0.25	−0.03	±0.18	−0.03	±0.13
Stressed **	0.03	±0.23	0.02	±0.15	0.05	±0.26	−0.03	±0.26	0.04	±0.24	0.07	±0.25	0.05	±0.12
Consistency ***	3.67	±0.79	3.51	±0.79	3.45	±0.77	3.54	±0.92	3.73	±0.85	3.69	±0.98	3.51	±0.85

* 0 – none, 1 – mild, 2 – moderate, 3 – severe.

** -1 – less than normal, 0 – normal, 1 – more than normal.

*** Bristol stool scale, 1 = hard, 7 = liquid.

b – significantly different to pre-treatment, (p < 0.05).

**P-values of volunteer diary changes.** A paired *t*-test was used to compare volunteer scores at different time points.

There were no significant changes in salivary sIgA concentrations (data not shown) or for volunteer dietary intakes when consuming the AXOS and placebo treatments (Table 6).

**Table 6:** Volunteer typical daily dietary intake whilst consuming the placebo and treatment bread products

	**Calories**	**Protein**	**Fat**	**Carbohydrate**	**Fibre**	**Non starch polysaccharides**	**Iron**
	**(kcal)**	**(g)**	**(g)**	**(g)**	**(g)**	**(g)**	**(mg)**
placebo	1796.0	68.1	53.8	227.5	19.8	14.9	12.9
treatment	±726.1	±31.2	±25.2	±110.5	±7.9	±6.0	±8.5
AXOS	1900.3	73.6	64.1	227.2	18.9	14.4	11.2
treatment	±767.5	±53.0	±27.4	±127.3	±5.7	±4.3	±3.9

(Average consumption from 4 consecutive days).

## Discussion

A double–blind, placebo-controlled, randomised cross-over study was undertaken to determine the potential benefits of AXOS-enriched bread on the gut. Bacteriology within faeces, faecal volatile fatty acid profiles and concentrations of sIgA in saliva, bowel habit and food intake of healthy volunteers was monitored throughout the feeding study.

During the study, 3 volunteers were withdrawn due to difficulties in eating 180 g bread daily. Based on the volunteer diaries, between 4 to 9 volunteers had difficulty in taking product in each of the periods. However, the breads did not lead to adverse symptoms and compliance was excellent with 99.24% of breads issued to the volunteers consumed. Volunteers were observed to be maintaining their diets throughout the study, with no significant changes in energy, protein, carbohydrate, fibre, non-starch polysaccharide, fat or iron consumption. The observation of increased stool number (stools/day) and an increased energetic feeling following placebo treatment (Table 5) was not observed following AXOS treatment. In the study of Cloetens et al., [24] AXOS extracted from wheat bran resulted in increased flatulence, this was not observed in the current study when AXOS was consumed at a lower dose within bread. Hence in the current study the AXOS-bread was well-tolerated.

Following AXOS treatment, a significant increase in faecal butyrate was observed (Table 4). Such an increase is regarded to be of benefit to the host, as butyrate is a major intestinal epithelial cell energy source, associated with potential anticancer activities observed as reducing cellular malignancy through stimulation of apoptosis in malignant cells [28]. Butyrate has also been observed to beneficially effect oxidative stress in the human colonic mucosa [43] and to induce immune-modulatory effects [44]. Furthermore, a trend for increased combined concentrations of acetate, propionate and butyrate, which are potentially beneficial to the host, was observed following AXOS-bread consumption compared to pre-AXOS treatment.

Protein fermentation is considered to be a non-beneficial process within the colon [45]. Following AXOS-bread consumption, a trend for reduced iso-valerate and reduced combined iso-butyrate, iso-valerate, valerate and caproate concentrations was observed (Table 4), which can therefore be viewed as a potentially positive effect as these organic acids are associated with protein fermentation [45]. This potentially beneficial modulation of fermentation of AXOS observed through VFA changes has been supported by previous research *in vitro*[22], within rats [27] and in humans [26]. It was therefore concluded that the AXOS-breads led to potential benefits following consumption.

Selective stimulation of indigenous beneficial gut genera is characteristic of a prebiotic [46]. Some potentially beneficial micro-organisms including *Lactobacillus paracasei, Bifidobacterium adolescentis and Bifidobacterium bifidum*[47-49] are able to breakdown AXOS. Previous studies have indicated that oat bran containing high levels of AX play a role in stimulating *Lactobacillus* and *Bifidobacterium*[50]. Moreover in research conducted by Cloetens et al. (2010) [24], AXOS (10 g/d) produced by partial enzymic hydrolysis of wheat AX led to a significant *Bifidobacterium* increase. Therefore, in the current study increases in numbers of faecal *Lactobacillus* and *Bifidobacterium* were expected. However, it was observed that levels of *Bifidobacterium* increased significantly from baseline following consumption of the control white breads (Table 3). It has previously been noted that white flour can be rich in fructans (up to 2.8%, [51]), and are largely available for fermentation in the large intestine [3] therefore it is likely that within the control white bread, with 180 g consumed daily, were sufficient fructans to have an effect on the faecal microbiota. The fructan content of rye flour has been observed to be as high as 4.5% [52]. The lack of observed bifidogenic effect, following consumption of the active breads, could therefore be attributable to the naturally occurring fructans within all bread products, thus masking any AXOS related effects. In a prebiotic dose related study [53], it was observed that a 10 g dose of galactooligosaccharide did not give rise to an enhanced bifidogenic effect when compared to a 5 g dose, it was therefore considered that above this threshold, the prebiotic dose did not determine the prebiotic effect. In terms of the current study this could explain that, although additionally 2.2 g AXOS were available within the treatment AXOS-bread, the required prebiotic dose for bifidogenicy had already been achieved in the control and placebo breads; thus any added effect was not apparent.

The bacteria numbers in the *Lactobacillus – Enterococcus* group were observed to significantly increase following the consumption of the placebo and AXOS-breads compared to pre-treatment breads (Table 3). Both of these breads were rye based, therefore it would seem that rye was influencing the microbiota. This has been supported by the use of rye grains commercially with lactobacilli for the production of sourdough [54,55]. This therefore indicates that rye may be considered as an appropriate substrate for lactobacilli.

Consumption of the placebo breads led to significant increases in numbers of bacteria in the *E. rectale* group, the *Roseburia* and the *Faecalibacterium prausnitzii* cluster compared to the pre-placebo treatment (Table 3). This was expected as AX, which would be found naturally occurring in the rye flour, is known to be non-digestible [56] and also stimulates the growth of organisms that possess microbial endoxylanase enzymes, such as bacteria within *E. rectaleRoseburia* groups [57] and *Faecalibacterium prausnitzii*. In previous human studies on AXOS, bacteria in the *E. rectale* group have not been stimulated following 10 g AXOS [24], thus this lower dose was not expected to stimulate growth of these microorganisms. Futhermore, *Roseburia* growth was not stimulated *in vitro* by AXOS [58], thus the AXOS bread may not be expected to stimulate growth of such microorganisms [59]. More research into this area would be required to determine this.

AXOS-breads were stimulatory to the microbiota, as observed through a significant increase in total bacteria compared to pre-AXOS treatment (Table 3). Previous intervention studies with a high dose (10 g/d) fructooligosaccharides have led to similar results [60]. An increase in the total bacteria numbers indicates that the AXOS breads have an impact on the microbiota. The butyrogenic effects then observed could relate to the direct activities of bacteria that have been stimulated, or from a cross feeding network taking place within the microbiota components [61] (Belenguer et al., 2006). There was no direct increase of bacteria within the *E. rectaleRoseburia* and *F. prausnitzii* groups which contain some known butyrate producers, however there are many other bacteria able to produce butyrate within the microbiota through both cross-feeding networks and also direct stimulation [62] (Louis and Flint 2007). An increase in butyrate is considered to be a beneficial effect to the host, therefore the AXOS breads lead to this endpoint, whilst the placebo breads did not.

Bacteroides constitutes a large proportion of the healthy adult gastrointestinal tract, that have an ability to adapt well to limited substrate availability [63]. In the current study, a significant increase in numbers of *Bacteroides – Prevotella* was observed following intervention with AXOS and placebo breads. This is in agreement with previous findings where both AXOS and AX have been observed to increase levels of *Bacteroides* spp. [19,59]. Bacteroides can grow on carbohydrates or on proteins; depending on the overall diet, proteolytic bacteroides are considered detrimental [64], whereas saccharolytic are not, because they produce potentially beneficial VFA [65]. In the current study as there is more carbohydrate likely to persist to the large intestine following the AXOS and placebo treatments, these changes are likely to be positive.

It has recently been observed that probiotics can lead to increased salivary secretory IgA levels [27]. In the current study, no changes in salivary sIgA were observed (Table 6). It is worth noting that sIgA has been seen to be elevated following a stressful work-session [66], and examination periods [67]. Furthermore, sIgA concentrations have declined in athletes following vigorous exercise [68]. Therefore, there are many other parameters that may be impacting on salivary sIgA results.

*In situ* enrichment of AXOS in breads provides an enhanced approach to straightforward fortification. This process utilises naturally occurring AX – furthermore xylanases are already used within the baking industry. The production of these breads may be subject to some variations, e.g. as rye and wheat composition may vary amid intrinsic and extrinsic factors. However, providing the level of AX within the grain is in excess of 2%, acceptable levels of AXOS generation would still be possible [18]. Therefore, it is unlikely that changes in grain will pose a problem in production of AXOS enriched breads.

Whilst shifts in microbial groups that are generally considered to be of benefit (bifidobacteria, lactobacilli) to the host were not observed following AXOS consumption, there were changes in the fermentation characteristics observed in terms of increased butyrate production and a trend for less protein fermentation markers. These results are important within a human population as similar shifts have been observed in animal studies and *in vitro*. Furthermore, the effect of such doses on the microbial community within humans has not been tested in such a population and whilst a (typically) beneficial bacterial shift could not be directly observed a fermentation shift showing positive potential was observed. This was not observed with the placebo products, therefore, although fermentation of the placebo was apparent, additional benefits from consumption of the AXOS breads – in terms of a fermentation shift are evident.

## Conclusions

AXOS-enriched breads were well tolerated and gave rise to a butyrogenic effect, which is of potential benefit to the consumer. Furthermore, a trend for reduced indicators of protein fermentation, with increased saccharolytic fermentation end products also suggests a beneficial shift in activities of bacteria. AXOS containing breads enabled enhanced bifidobacterial numbers that existed following the control bread treatment to be maintained. It is likely that a high level of bifidobacteria, following control bread consumption contributed to the stable population seen during the consumption of placebo or AXOS-breads, thus further bifidogenic effects of the treatment breads could not be elucidated. An increase in total bacteria following consumption of AXOS-bread showed them to be stimulatory to the faecal microbiota; furthermore both AXOS and placebo breads led to increases in the potentially beneficial lactobacilli group. The effects on fermentation end-products, that were not observed following the placebo breads, indicate that AXOS-bread consumption elicited a potentially beneficial shift in fermentation characteristics.

## Competing interests

This intervention study was made possible by the financial support of Puratos Group, who also provided the bread products used within the study. Isabel Trogh and Filip Arnaut are both employees of Puratos Group. There are no other competing interests.

## Authors’ contributions

The study was designed by GRG, IT and FA. The intervention study, recruitment and statistical analysis was executed by GEW. Data acquisition was carried out by GEW and CL. GRG and GEW interpreted the data. GEW, GRG, CL, IT and AF were all involved in writing the manuscript. All authors read and approved the final manuscript

## References

[B1] Dubert-Ferrandon A Newburg DS Walker WA Part 2-Prebiotics: New medicines for the colon, health benefits Nutr Today 2009 44 85 91 10.1097/NT.0b013e31819df7bc

[B2] Szilagyi A Prebiotics or probiotics for lactose intolerance: a question of adaptation Am J Clin Nutr 1999 70 1 105 106 10393148 10.1093/ajcn/70.1.105

[B3] Evenepoel P Meijers BK Bammens BR Verbeke K Uremic toxins originating from colonic microbial metabolism Kidney Int 2009 144 Suppl 12 19 10.1038/ki.2009.402 19946322

[B4] Ooi L Liong M Cholesterol-lowering effects of probiotics and prebiotics: a review of in vivo and in vitro findings Int J Mol Sci 2010 11 2499 2522 10.3390/ijms11062499 20640165 PMC2904929

[B5] Abrams SA Griffin IJ Hawthorne KM Liang L Gunn SK Darlington G Ellis KJ A combination of prebiotic short and long-chain inulin-type fructans enhances calcium absorption and bone mineralization in young adolescents Am J Clin Nutr 2005 82 471 476 16087995 10.1093/ajcn.82.2.471

[B6] Burns AJ Rowland IR Antigenotoxicity of probiotics and prebiotics on faecal water-induced DNA damage in human colon adenocarcinoma cells Muta Res 2004 551 233 243 10.1016/j.mrfmmm.2004.03.010 15225596

[B7] Klinder A Förster A Caderni G Femia AP Pool-Zobel BL Faecal water genotoxicity is predictive of tumor preventive activities by inulin-like oligofructoses, probiotics (Lactobacillus rhamnosus and Bifidobacterium lactis) and their synbiotic combination Nutr Cancer 2004 49 144 155 10.1207/s15327914nc4902_5 15489207

[B8] Pool-Zobel BL Inulin-type fructans and reduction in colon cancer risk: review of experimental and human data Br J Nutr 2005 93 Suppl 73 90 15877900 10.1079/bjn20041349

[B9] Silk DB Davis A Vulevic J Tzortzis G Gibson GR Cl inical trial: the effects of a trans-galactooligosaccharide prebiotic on faecal microbiota and symptoms in irritable bowel syndrome Aliment Pharmacol Ther 2009 29 508 518 10.1111/j.1365-2036.2008.03911.x 19053980

[B10] Gibson GR Probiotics and prebiotics and their function Funct Nutr 2003 2 11 13

[B11] Roberfroid MB Prebiotics and probiotics: are they functional foods? Am J Clin Nutr 2000 71 Suppl 6 1682 1687 10.1093/ajcn/71.6.1682S 10837317

[B12] Tuohy KM Kolida S Lustenberger AM Gibson GR The prebiotic effects of biscuits containing partially hydrolysed guar gum and fructo-oligosaccharides – a human volunteer study Br J Nutr 2001 86 341 348 10.1079/BJN2001394 11570986

[B13] Costabile A Kolida S Klinder A Gietl E Bäuerlein M Frohberg C Landschütze V Gibson GR A double-blind, placebo-controlled, cross-over study to establish the bifidogenic effect of a very-long-chain inulin extracted from globe artichoke (Cynara scolymus) in healthy human subjects Br J Nutr 2010 104 1007 1017 10.1017/S0007114510001571 20591206

[B14] Kolida S Meyer D Gibson GR A double-blind placebo-controlled study to establish the bifidogenic dose of inulin in healthy humans Eu J Clin Nutr 2007 61 1189 1195 10.1038/sj.ejcn.1602636 17268410

[B15] Ramnani P Gaudier E Bingham M Bruggen P Van Tuohy KM Gibson GR Prebiotic effect of fruit and vegetable shots containing Jerusalem artichoke inulin: a human intervention study Br J Nutr 2010 104 233 240 10.1017/S000711451000036X 20187995

[B16] Vulevic J Drakoularakou A Yaqoob P Tzortzis G Gibson GR Modulation of the fecal microflora profile and immune function by a novel trans-galactooligosaccharide mixture (B-GOS) in healthy elderly volunteers Am J Clin Nutr 2008 88 1438 1446 18996881 10.3945/ajcn.2008.26242

[B17] Courtin CM Delcour JA Arabinoxylans and Endoxylanases in Wheat Flour Bread-making J Cereal Sci 2002 35 225 243 10.1006/jcrs.2001.0433

[B18] Van Haesendonck IPH Broekaert WF Georis J Delcour J Courtin C Filip A Bread with increased arabinoxylo-oligosaccharide content 2008 Publication no Geneva: WIPO Publication no. WO08087167

[B19] Kamp JWVD Asp NG Jones JM Schaafsma G Dietary Fibre — bio-active carbohydrates for food and feed 2004 The Netherlands: Wageningen Academic Publishers

[B20] Van Craeyveld V Swennen K Dornez E Van de Wiele T Marzorati M Verstraete W Delaedt Y Onagbesan O Decuypere E Buyse J De Ketelaere B Broekaert WF Delcour JA Courtin CM Structurally different wheat-derived arabinoxylooligosaccharides have different prebiotic and fermentation properties in rats Am J Clin Nutr 2008 138 2348 2355 10.3945/jn.108.094367 19022956

[B21] Hughes SA Shewry PR Li L Gibson GR Sanz ML Rastall RA In vitro fermentation by human fecal microflora of wheat arabinoxylans J Agric Food Chem 2007 55 4589 4595 10.1021/jf070293g 17488118

[B22] Sanchez JI Marzorati M Grootaert C Baran M Van Craeyveld V Courtin CM Broekaert WF Delcour JA Verstraete W Van de Wiele T Arabinoxylan-oligosaccharides (AXOS) affects the protein/carbohydrate fermentation balance and microbial population dynamics of the simulator of human intestinal microbial ecosystem Microb Biotechnol 2009 2 101 113 10.1111/j.1751-7915.2008.00064.x 21261885 PMC3815425

[B23] Courtin CM Swennen K Broekaert WF Swennen Q Buyse J Decuypere E Michiels CW De Ketelaere B Delcour JA Effects of dietary inclusion of xylooligosaccharides, arabinoxylooligosaccharides and soluble arabinoxylan on the microbial composition of caecal contents of chickens J Sci Food Agric 2008 88 2517 2522 10.1002/jsfa.3373

[B24] Cloetens L Broekaert WF Delaedt Y Ollevier F Courtin CM Delcour JA Rutgeerts P Verbeke K Tolerance of arabinoxylan-oligosaccharides and their prebiotic activity in healthy subjects: a randomised, placebo-controlled cross-over study Br J Nutr 2010 103 703 713 10.1017/S0007114509992248 20003568

[B25] Eeckhaut V Van Immerseel F Dewulf J Pasmans F Haesebrouck F Ducatelle R Courtin CM Delcour JA Broekaert WF Arabinoxylooligosaccharides from wheat bran inhibit Salmonella colonization in broiler chickens Poult Sci 2008 87 2329 2334 10.3382/ps.2008-00193 18931184

[B26] Cloetens L De Preter V Swennen K Broekaert WF Courtin CM Delcour JA Rutgeerts P Verbeke K Dose–response effect of arabinoxylooligosaccharides on gastrointestinal motility and on colonic bacterial metabolism in healthy volunteers J Am Coll Nutr 2008 27 512 518 18978172 10.1080/07315724.2008.10719733

[B27] Van Craeyveld V Swennen K Dornez E Van de Wiele T Marzorati M Verstraete W Delaedt Y Onagbesan O Decuypere E Buyse J De Ketelaere B Broekaert WF Delcour JA Courtin CM Structurally different wheat-derived arabinoxylooligosaccharides have different prebiotic and fermentation properties in rats J Nutr 2008 138 2348 2355 10.3945/jn.108.094367 19022956

[B28] Dronamraju SS Coxhead JM Kelly SB Mathers JC Differential antineoplastic effects of butyrate in cells with and without a functioning DNA mismatch repair Nutr Cancer 2010 62 105 115 20043265 10.1080/01635580903191486

[B29] Collins JK Dunne C Murphy L Morrissey D O'Mahony L O'Sullivan E Fitzgerald G Kiely B O'Sullivan GC Daly C Marteau P Shanahan F A randomized controlled trial of a probiotic Lactobacillus strain in healthy adults: assessment of its delivery, transit and influence on microbial flora and enteric immunity Microb Ecol Health Dis 2002 14 81 89 10.1080/08910600260081720

[B30] Courtin CM Van den Broeck H Delcour JA Determination of reducing end sugar residues in oligo- and polysaccharides by gas–liquid chromatography J Chromatogr 2000 866 97 104 10.1016/S0021-9673(99)01064-X 10681013

[B31] Damen B Cloetens L Broekaert WF François I Lescroart O Trogh I Arnaut F Welling GW Wijffels J Delcour JA Verbeke K Courtin CM Consumption of breads containing in situ-produced arabinoxylan oligosaccharides alters gastrointestinal effects in healthy volunteers J Nutr 2012 142 470 477 10.3945/jn.111.146464 22298569

[B32] Martín-Peláez S Gibson GR Martín-Orúe SM Klinder A Rastall RA La Ragione RM Woodward MJ Costabile A In vitro fermentation of carbohydrates by porcine faecal inocula and their influence on Salmonella Typhimurium growth in batch culture systems FEMS Microbiol Ecol 2008 66 3 608 619 10.1111/j.1574-6941.2008.00610.x 19049655

[B33] Harmsen HJM Wildeboer-Veloo ACM Grijpstra J Knol J Degener JE Welling GW Development of 16 S rRNA-based probes for the Coriobacterium group and the Atopobium cluster and their application for enumeration of Coriobacteriaceae in human faeces from volunteers of different age groups Appl Environ Microbiol 2000 66 4523 4527 10.1128/AEM.66.10.4523-4527.2000 11010909 PMC92335

[B34] Manz W Amann R Ludwig W Vancanneyt M Schleifer KH Application of a suite of 16 S rRNA-specific oligonucleotide probes designed to investigate bacteria of the phylum cytophaga-flavobacter-bacteroides in the natural environment Microbiol 1996 142 1097 1106 10.1099/13500872-142-5-1097 8704951

[B35] Langendijk PS Schut F Jansen GJ Raangs GC Kamphuis GR Wilkinson MH Welling GW Quantitative fluorescence in situ hybridization of Bifidobacterium spp. with genus-specific16S rRNA-targeted probes and its application in faecal samples Appl Environ Microbiol 1995 61 3069 3075 7487040 10.1128/aem.61.8.3069-3075.1995 PMC167584

[B36] Franks AH Harmsen HJM Raangs GC Jansen GJ Schut F Welling GW Variations of bacterial populations in human faeces measured by fluorescence in situ hybridization with group-specific 16 S rRNA-targeted oligonucleotide probes Appl Environ Microbiol 1998 64 3336 3345 9726880 10.1128/aem.64.9.3336-3345.1998 PMC106730

[B37] Harmsen HJM Elfferich P Schut F Welling GW A16S rRNA-targeted probe for detection of lactobacilli and enterococci in faecal samples by fluorescence in situ hybridization Microb Ecol Health Dis 1999 11 3 12 10.1080/089106099435862

[B38] Poulsen LK Lan F Kristensen CS Hobolth P Molin S Krogfelt KA Spatial distribution of Escherichia coli in the mouse large intestine inferred from rRNA in situ hybridization Infect Immun 1994 62 5191 5194 7927805 10.1128/iai.62.11.5191-5194.1994 PMC303247

[B39] Walker AW Duncan SH McWilliam Leitch EC, Child MW, Flint HJ: pH and peptide supply can radically alter bacterial populations and short-chain fatty acid ratios within microbial communities from the human colon Appl Environ Microbiol 2005 71 3692 3670 10.1128/AEM.71.7.3692-3700.2005 16000778 PMC1169066

[B40] Suau A Rochet V Sghir A Gramet G Brewaeys S Sutren M RigottierGois L Dore J Fusobacterium prausnitzii and related species represent a dominant group within the human fecal flora Systems Appl Microbiol 2001 24 139 145 10.1078/0723-2020-00015 11403393

[B41] Daims H Brühl A Amann R Schleifer KH Wagner M The domain-specific probe EUB338 is insufficient for the detection of all Bacteria: development and evaluation of a more comprehensive probe set Syst Appl Microbiol 1999 22 434 444 10.1016/S0723-2020(99)80053-8 10553296

[B42] Richardson AJ Calder AG Stewart CS Smith A Simultaneous determination of volatile and non-volatile acid fermentation products of anaerobes by capillary gas chromatography Lett Appl Microbiol 1989 9 5 8 10.1111/j.1472-765X.1989.tb00278.x

[B43] Hamer HM Jonkers DM Bast A Vanhoutvin SA Fischer MA Kodde A Troost FJ Venema K Brummer RJ Butyrate modulates oxidative stress in the colonic mucosa of healthy humans Clin Nutr 2009 28 88 93 10.1016/j.clnu.2008.11.002 19108937

[B44] Böcker U Nebe T Herweck F Holt L Panja A Jobin C Rossol SB Sartor R Singer MV: Butyrate modulates intestinal epithelial cell-mediated neutrophil migration Clin Exp Immunol 2003 131 53 60 10.1046/j.1365-2249.2003.02056.x 12519386 PMC1808611

[B45] Mortensen PB Clausen MR Bonnén H Hove H Holtug K Colonic fermentation of ispaghula, wheat bran, glucose, and albumin to short-chain fatty acids and ammonia evaluated in vitro in 50 subjects PEN J Parenter Enteral Nutr 1992 16 433 439 10.1177/0148607192016005433 1331553

[B46] Gibson GR Scott KP Rastall RA Tuohy KM Hotchkiss A Dubert-Ferrandon A Gareau M Murphy EF Saulnier D Loh G Macfarlane S Delzenne N Ringel Y Kozianowski G Dickmann R Lenoir-Wijnkook I Walker C Buddington R Dietary prebiotics: current status and new definition IFIS Func Foods Bull 2010 7 1 19

[B47] Crittenden R Karppinen S Ojanen S Tenkanen M Fagerstrom R Mättö J Saarela M Mattila-Sandholm T Poutanen K In vitro fermentation of cereal dietary fibre carbohydrates by probiotic and intestinal bacteria J Sci Food Agric 2002 82 781 789 10.1002/jsfa.1095

[B48] Kontula P Suihko ML Suortti T Tenkanen M Mattila-Sandholm T von Wright A The isolation of lactic acid bacteria from human colonic biopsies after enrichment on lactose derivatives and rye arabinoxylo-oligosaccharides Food Microbiol 2000 17 13 22 10.1006/fmic.1999.0268

[B49] Van Laere KMJ Hartemink R Bosveld M Schols HA Voragen AGJ Fermentation of plant cell wall derived polysaccharides and their corresponding oligosaccharides by intestinal bacteria J Agric Food Chem 2000 48 1644 1652 10.1021/jf990519i 10820072

[B50] Jaskari J Kontula P Siitonen A Jousimies-Somer H Mattila-Sandholm T Poutanen K Oat beta-glucan and xylan hydrolysates as selective substrates for Bifidobacterium and Lactobacillus strains Appl Microbiol and Biotech 1998 49 175 181 10.1007/s002530051155 9534257

[B51] Venter CS Prebiotics: an update JFECS 2007 35 17 25

[B52] Karppinen S Myllymäki O Forssell P Poutanen K Fructan content of rye and rye products Cereal Chem 2003 80 168 171 10.1094/CCHEM.2003.80.2.168

[B53] Davis LM Martínez I Walter J Hutkins R A dose dependent impact of prebiotic galactooligosaccharides on the intestinal microbiota of healthy adults Intl J Food Microbiol 2010 44 285 292 10.1016/j.ijfoodmicro.2010.10.007 21059476

[B54] Muller MRA Ehrmann M Vogel RF Lactobacillus frumenti sp. nov., a new lactic acid bacterium isolated from rye-bran fermentations with a long fermentation period Int J Syst Evol Micr 2000 50 2127 2133 10.1099/00207713-50-6-2127 11155988

[B55] Vogel RF Böcker G Stolz P Ehrmann M Fanta D Ludwig W Pot B Kersters K Schleifer KH Hammes WP Identification of lactobacilli from sourdough and description of Lactobacillus pontis sp. nov Int J Syst Bacteriol 1994 44 223 229 10.1099/00207713-44-2-223 8186088

[B56] Glitso LV Gruppen H Schols HA Hojsgaard S Sandstrom B Knudsen KEB Degradation of rye arabinoxylans in the large intestine of pigs J Sci Food Agric 1999 79 61 969

[B57] Chassard C Goumy V Leclerc M Del’homme C Bernalier-Donadille A Characterization of the xylan-degrading microbial community from human faeces FEMS Microbiol Ecol 2007 61 121 131 10.1111/j.1574-6941.2007.00314.x 17391327

[B58] Broekaert WF Courtin CM Verbeke K Van de Wiele T Verstraete W Delcour JA Prebiotic and other health-related effects of cereal-derived arabinoxylans, arabinoxylan-oligosaccharides, and xylooligosaccharides Crit Rev Food Sci Nutr 2011 51 178 194 10.1080/10408390903044768 21328111

[B59] Rigottier-Gois L Rochet V Garrec N Suau A Doré J Enumeration of Bacteroides species in human faeces by fluorescent in situ hybridisation combined with flow cytometry using 16 S rRNA probes Syst Appl Microbiol 2003 26 110 118 10.1078/072320203322337399 12747418

[B60] Grootaert C Van Den Abbeele P Marzorati M Broekaert WF Courtin CM Delcour JA Verstraete W Van De Wiele T Comparison of prebiotic effects of arabinoxylan oligosaccharides and inulin in a simulator of the human intestinal microbial ecosystem FEMS Microbiol Ecol 2009 69 231 242 10.1111/j.1574-6941.2009.00712.x 19508502

[B61] Belenguer A Duncan SH Calder AG Holtrop G Louis P Lobley GE Flint HJ Two routes of metabolic cross-feeding between Bifidobacterium adolescentis and butyrate-producing anaerobes from the human gut Appl Environ Microbiol 2006 72 3593 3599 10.1128/AEM.72.5.3593-3599.2006 16672507 PMC1472403

[B62] Louis P Flint HJ Diversity, metabolism and microbial ecology of butyrate-producing bacteria from the human large intestine FEMS Microbiol Lett 2009 294 1 8 10.1111/j.1574-6968.2009.01514.x 19222573

[B63] Macfarlane GT Hay S Macfarlane S Gibson GR Effect of different carbohydrates on growth, polysaccharidase and glycosidase production by Bacteroides ovatus, in batch and continuous culture J Appl Bacteriol 1990 68 179 187 10.1111/j.1365-2672.1990.tb02564.x 2318746

[B64] Macfarlane GT Cummings JH Allison C Protein degradation by human intestinal bacteria J Gen Microbiol 1986 132 1647 1656 3543210 10.1099/00221287-132-6-1647

[B65] Mortensen PB Clausen MR Short-chain fatty acids in the human colon: relation to gastrointestinal health and disease Scand J Gastroenterol 1996 216 Suppl 132 148 10.3109/00365529609094568 8726286

[B66] Zeier H Brauchli P Joller-Jemelka HI Effects of work demands on immunoglobulin A and cortisol in air traffic controllers Biol Psychol 1996 42 413 423 10.1016/0301-0511(95)05170-8 8652756

[B67] Matos-Gomes N Katsurayama M Makimoto FH Santana LL Paredes-Garcia E Becker MA Dos-Santos MC Psychological stress and its influence on salivary flow rate, total protein concentration and IgA, IgG and IgM titers Neuroimmunomodulation 2010 17 396 404 10.1159/000292064 20516721

[B68] Moreira A Arsati F Cury PR Franciscon C Simões AC de Oliveira PR de Araújo VC The impact of a 17-day training period for an international championship on mucosal immune parameters in top-level basketball players and staff members Eur J Oral Sci 2008 116 431 437 10.1111/j.1600-0722.2008.00558.x 18821985

